# Visible Lights Combined with Photosensitizing Compounds Are Effective against *Candida albicans* Biofilms

**DOI:** 10.3390/microorganisms9030500

**Published:** 2021-02-26

**Authors:** Priyanka Bapat, Gurbinder Singh, Clarissa J. Nobile

**Affiliations:** 1 Department of Molecular and Cell Biology, School of Natural Science, University of California, Merced, CA 95343, USA; pbapat@ucmerced.edu (P.B.); gsingh57@ucmerced.edu (G.S.); 2 Quantitative and Systems Biology Graduate Program, University of California, Merced, CA 95343, USA; 3 Health Sciences Research Institute, University of California, Merced, CA 95343, USA

**Keywords:** *Candida albicans*, biofilms, red, green, and blue (RGB) visible lights, photodynamic therapy, photosensitizing compounds, reactive oxygen species (ROS), non-drug therapeutic strategies, non-drug antifungal strategies

## Abstract

Fungal infections are increasing in prevalence worldwide, especially in immunocompromised individuals. Given the emergence of drug-resistant fungi and the fact that there are only three major classes of antifungal drugs available to treat invasive fungal infections, there is a need to develop alternative therapeutic strategies effective against fungal infections. *Candida albicans* is a commensal of the human microbiota that is also one of the most common fungal pathogens isolated from clinical settings. *C. albicans* possesses several virulence traits that contribute to its pathogenicity, including the ability to form drug-resistant biofilms, which can make *C. albicans* infections particularly challenging to treat. Here, we explored red, green, and blue visible lights alone and in combination with common photosensitizing compounds for their efficacies at inhibiting and disrupting *C. albicans* biofilms. We found that blue light inhibited biofilm formation and disrupted mature biofilms on its own and that the addition of photosensitizing compounds improved its antibiofilm potential. Red and green lights, however, inhibited biofilm formation only in combination with photosensitizing compounds but had no effects on disrupting mature biofilms. Taken together, these results suggest that photodynamic therapy may be an effective non-drug treatment for fungal biofilm infections that is worthy of further exploration.

## 1. Introduction

Fungi cause a wide range of diseases in humans ranging from superficial skin to life-threatening disseminated infections, especially in immunocompromised and critically ill individuals [1]. *Candida albicans* is a common fungus that typically resides as a benign commensal member of the human microbiota, colonizing the skin and mucosal surfaces of healthy humans [2]. It is also an opportunistic pathogen that can cause both superficial skin and mucosal infections as well as severe systemic infections under permissive host environmental conditions [3,4]. *C. albicans* has multiple virulence mechanisms that contribute to its pathogenicity, including the ability to form physically recalcitrant and drug-resistant biofilms, that can make *C. albicans* infections particularly challenging to treat [5].

**Figure 1:**
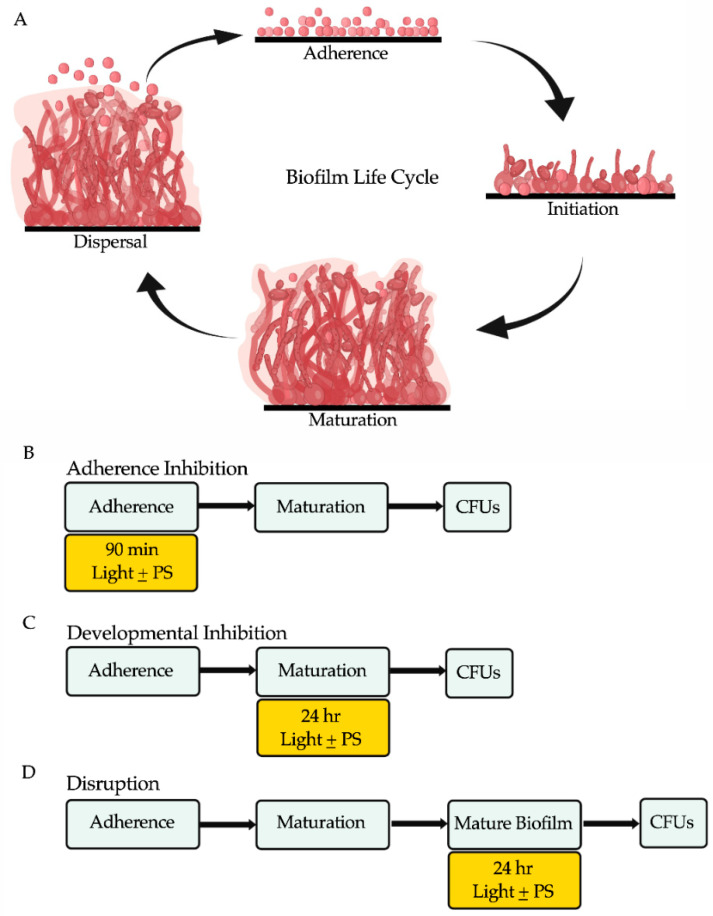
The *C. albicans* biofilm life cycle and the biofilm assays used in this study to assess the antibiofilm properties of visible lights and photosensitizing compounds. (**A**) The *C. albicans* biofilm life cycle occurs in four sequential stages: adherence, initiation, maturation, and dispersal. In the adherence stage, planktonic yeast-form cells adhere to a surface. In the initiation stage, the yeast-form cells proliferate forming an anchoring basal cell layer and begin to differentiate into hyphal and pseudohyphal cells. In the maturation stage, the hyphal cells elongate, and a protective extracellular matrix surrounds the cells. In the dispersal stage, yeast-form cells are released from the biofilm and the life cycle repeats. (**B**) Overview of the adherence inhibition biofilm assay, where the visible light of interest with (+) or without (−) the photosensitizing compound (PS) of interest were present during the 90-min adherence stage of biofilm formation. (**C**) Overview of the developmental inhibition biofilm assay, where the visible light of interest with (+) or without (−) the PS of interest were present during the 24 h maturation stage of biofilm formation. (**D**) Overview of the disruption biofilm assay, where the visible light of interest with (+) or without (−) the PS of interest were present for an additional 24 h on a mature (24-h) biofilm. Colony forming units (CFUs) were measured to determine viable cell counts at the end of each biofilm assay. This figure was creating using BioRender.com.

Biofilms are communities of adherent microbial cells encased in extracellular matrices that are often resistant and/or tolerant to antimicrobial agents and the host immune response [6,7,8]. The *C. albicans* biofilm life cycle occurs in four sequential stages: adherence, initiation, maturation, and dispersal (Figure 1A). In the adherence stage, planktonic (free-floating) yeast-form cells adhere to biotic surfaces (e.g., mucosal layers and epithelial cell layers) or abiotic surfaces (e.g., catheters, heart valves, and dentures) [9]. In the initiation stage, the yeast-form cells proliferate to form an anchoring basal cell layer and begin to differentiate into hyphal and pseudohyphal cells. In the maturation stage, the hyphal cells elongate and a protective extracellular matrix that is composed of proteins, carbohydrates, nucleic acids, and lipids surrounds the cells within the biofilm. In the dispersal stage, which completes the *C. albicans* biofilm life cycle, yeast-form cells are released from the biofilm, where they can repeat the biofilm life cycle by forming biofilms at secondary sites in the host or can enter the bloodstream to cause life-threatening systemic infections [3,4,8].

Antifungal drugs are the most commonly used therapeutic agents for treating fungal infections [10]. Only three major classes of antifungal drugs (the polyenes, azoles, and echinocandins) are currently used to treat invasive fungal infections in humans, and it has been a challenge to develop new and effective antifungal drugs, especially with efficacy against biofilms [11,12,13,14]. Existing antifungal drugs often have significant side effects in humans, causing toxicity to the liver, kidneys, and central nervous system [15,16]. Additionally, some *Candida* clinical isolates are naturally resistant and/or tolerant to antifungal drugs or can develop resistance over time, further reducing treatment efficacy [17,18]. The paucity of effective antifungal drugs with low toxicity to humans, combined with an increase in antifungal drug resistance in *Candida* clinical isolates, has prompted the search for alternative non-drug therapeutic strategies to treat fungal infections [19].

Photodynamic therapy has been used over the last 40 years to treat oncologic skin conditions, such as basal cell carcinoma and actinic keratosis [20,21], and more recently to treat benign skin conditions, such as acne vulgaris and viral warts [22]. Currently, and in light of the emergence of drug resistant infections in the clinic, photodynamic therapy as a non-drug antimicrobial strategy has been gaining considerable scientific interest [23,24,25,26]. Photodynamic therapy relies on a light source, a non-toxic photosensitizing compound that can absorb and transfer electrons after light absorption, and molecular oxygen that acts as an electron acceptor [23]. The typical output of photodynamic therapy is reactive oxygen species (ROS) (e.g., singlet oxygen, hydroxyl radicals, and superoxide anions) that are produced when the photosensitizing compound is excited by light; these ROS can then have cytotoxic effects on the targeted cells, such as cancer cells and microbial cells [27,28]. Unlike traditional antimicrobial drugs, photodynamic therapy as an antimicrobial strategy would affect multiple non-specific microbial targets simultaneously, making it unlikely for resistance to be developed. Based on its fundamental mechanisms of action, photodynamic therapy could be a clinically useful non-drug antimicrobial therapeutic strategy that is worthy of further exploration.

The visible light spectrum can be broadly divided into red (620–700 nm), green (500–560 nm), and blue (400–490 nm) wavelengths [23,24,29,30], where several discreet wavelengths within each spectrum have been shown to display antimicrobial properties [29,31,32,33]. To date, of the visible lights, blue light has been the most studied for its antimicrobial properties, where it has been shown to effectively kill pathogenic bacteria and fungi in vitro, including drug-resistant bacteria in both planktonic and biofilm forms [34,35,36,37,38,39,40,41,42,43,44,45,46,47]. Comparatively, the antimicrobial properties of red and green lights have been much less studied to date [29,48,49,50,51].

Although the use of lights in the visible spectrum can have antimicrobial effects on targeted microbial cells on their own, likely by generating ROS through the photoexcitation of naturally occurring photosensitizing compounds (e.g., flavoproteins and porphyrins) [28,40], the combined antimicrobial effects of visible lights with exogenous synthetic photosensitizing compounds have been shown to significantly increase the generation of ROS in vitro [26,42,52,53]. There are many non-toxic synthetic photosensitizing compounds that have been developed to date [54,55,56,57], but in this study we focus on the classic and commonly used photosensitizing compounds new methylene blue, toluidine blue O, and rose bengal (Figure S1). New methylene blue and toluidine blue O are structurally similar phenothiazinium salts absorbing between 600 and 660 nm, while rose bengal is a xanthene salt absorbing between 500 and 550 nm [42,47,52,58,59].

Prior work on *C. albicans* has shown that the combination of blue light with rose bengal reduced *C. albicans* cell viability in both planktonic and biofilm forms [59]. Additionally, a combination of blue light with toluidine blue O inhibited *C. albicans* biofilm formation [47]. For red light, in combination with new methylene blue, *C. albicans* cell viability in the planktonic form was reduced [60]. Finally, for green light in combination with rose bengal, *C. albicans* cell viability in both planktonic and biofilm forms was reduced [61]. To our knowledge, no studies to date have compared different visible lights alone or in combination with photosensitizing compounds to assess their efficacies at inhibiting and disrupting *C. albicans* biofilms at different stages of biofilm formation. Our study assesses the effects of these lights at the adherence stage of biofilm formation, throughout the course of biofilm formation, and on mature biofilms. In addition, our study includes *C. albicans* strains of different genetic backgrounds, which is important for understanding the real-world utility of antimicrobial photodynamic therapy in clinical settings.

In this study, we examined and compared the effects of red, green, and blue visible lights alone and in combination with the classic and commonly used photosensitizing compounds new methylene blue, toluidine blue O, and rose bengal to assess their efficacies at inhibiting *C. albicans* biofilm formation and at disrupting mature *C. albicans* biofilms. We found that blue light inhibited biofilm formation and disrupted mature biofilms on its own and that the addition of photosensitizing compounds improved its antibiofilm potential. Red and green lights, however, inhibited biofilm formation only in combination with photosensitizing compounds, but had no effects on disrupting mature biofilms.

## 2. Materials and Methods

### 2.1. Strains and Media

All experiments were performed using the wildtype *C. albicans* strain SN250 [62]. The results using SN250 were validated using the *C. albicans* clinical isolates SC5314 [63] and Strain #0761 (AR0761) (Centers for Disease Control and Prevention (CDC) AR Isolate Bank, Drug Resistance *Candida* species panel; https://wwwn.cdc.gov/ARIsolateBank/ (access on 5 February 2021). *C. albicans* cells were recovered from −80 °C glycerol stocks for two days at 30 °C on yeast extract peptone dextrose (YPD) agar plates (1% yeast extract (Thermo Fisher Scientific, Catalog #211929), 2% Bacto peptone (Gibco, Catalog #211677), 2% dextrose (Fisher Scientific, Catalog #D16-3), and 2% agar (Criterion, Catalog #89405-066)). Overnight cultures were grown for ~15 h at 30 °C, shaking at 225 rpm in YPD liquid medium (1% yeast extract (Thermo Fisher Scientific, Catalog #211929), 2% Bacto peptone (Gibco, Catalog #211677), and 2% dextrose (Fisher Scientific, Catalog #D16-3)). All biofilm assays were performed using Spider medium (10 g/L nutrient broth (VWR, Catalog #89405-794), 10 g/L mannitol (Alfa Aesar, Catalog #A14030), 4 g/L K_2_PO_4_ (Fisher Scientific, Catalog #P290-212)), at pH 7.2.

### 2.2. Light Sources and Photosensitizing Compounds

A red light-emitting diode (LED) light source (ABI LED lighting, Catalog #GR-PAR38-26W-RED, 26-Watt 620–630 nm, outputting 176 J/cm^2^), a green LED light source (ABI LED lighting, Catalog #GR-PAR38-24W-520NM, 24-Watt 520–530 nm, outputting 204 J/cm^2^), and a blue LED light source (ABI LED lighting, Catalog #GR-PAR38-24W-BLU, 24-Watt 450 nm, outputting 240 J/cm^2^) were placed 8 inches from the biofilm wells and used as indicated in the biofilm assays. Average LED light intensity measurements for each light source at a distance of 8 inches away from the biofilm assay plates were 6500 lux for red light, 6700 lux for green light, and 5900 lux for blue light.

The photosensitizing compounds new methylene blue (Sigma Aldrich, Catalog #B-4631), toluidine blue O (Sigma Aldrich, Catalog #T3260) and rose bengal (Sigma Aldrich, Catalog #198250) were used as indicated in the biofilm assays. The photosensitizing compounds were dissolved in phosphate buffered saline (PBS) (HyClone, Catalog #16777-252) at a stock concentration of 10 mM and diluted to a working concentration of 400 μM in Spider medium, which was used to grow the biofilms. Stocks of the photosensitizing compounds were prepared fresh every two weeks, filter sterilized, and stored at 4 °C in the dark.

### 2.3. Biofilm Assays

The adherence inhibition, developmental inhibition, and disruption biofilm assays were performed as described previously [64,65], except that instead of taking optical density readings at the end of the biofilm assays, we measured colony forming units (CFUs) to assess the efficacies of the visible lights with or without photosensitizing compounds at reducing *C. albicans* viable cell counts from the biofilms. This modification was made because the photosensitizing compounds on their own elevated optical density readings by absorbing light, and as such, optical density readings did not accurately reflect biofilm growth or thickness.

In brief, biofilms were grown in triplicate on the bottoms of sterile flat-bottomed 12-well non-tissue culture treated polystyrene plates (Corning, Catalog #351143). The 12-well plates were seeded at a final OD_600_ of 0.5 in a final volume of 2 mL Spider medium and grown for 90 min at 37 °C with shaking at 250 rpm in an ELMI shaker (M2 Scientifics, Catalog #ELMI-TRMS04). After the initial 90 min adherence period, the wells were gently washed with PBS and fresh Spider medium was added to each well. The plates were sealed with breathable sealing membranes (Sigma Aldrich, Catalog #Z380059) and grown at 37 °C with shaking at 250 rpm in an ELMI shaker for 24 h. For the adherence inhibition biofilm assay, the biofilms were exposed to red, green, or blue visible lights with or without a photosensitizing compound during the 90 min adherence stage of biofilm formation (Figure 1B). For the developmental inhibition biofilm assay, the biofilms were exposed to red, green, or blue visible lights with or without a photosensitizing compound throughout the first 24 h of biofilm growth, but not during the initial 90-min adherence stage (Figure 1C). For the disruption biofilm assay, medium was removed from each well containing a mature 24-h-old biofilm, fresh Spider medium was added to each well, the plates were re-sealed, and the mature biofilms were exposed to red, green, or blue visible lights with or without a photosensitizing compound for an additional 24 h (Figure 1D). The 12-well plates were divided such that half of one plate was exposed to the light of interest and the other half was covered with foil and served as a no light control.

### 2.4. Determination of Colony Forming Units (CFUs) from Biofilms

CFU determinations from biofilms were performed as previously described [64,65]. Briefly, biofilms were scraped from the bottoms of each well of a 12-well plate using a sterile spatula, vigorously vortexed, serially diluted in PBS, and plated onto YPD agar plates. The plates were incubated at room temperature for 3 days and colonies were counted to determine CFUs/mL. Statistical significance was determined using Student’s unpaired two-tailed t-test, assuming unequal variance.

We note that we do not recommend measuring the metabolic reduction of the tetrazolium salt reagent 2,3-bis-(2-methoxy-4-nitro-5-sulfophenyl)-2H-tetrazolium-5-carboxanilide (XTT) as a method to assess metabolic activity in the presence of photosensitizing compounds, because the photosensitizing compounds on their own (as is the case with the photosensitizing compounds used in our study) can elevate optical density readings by absorbing light in this colorimetric assay, and as such the XTT assay would not accurately reflect metabolic activity after treatment.

### 2.5. Viability Staining of Biofilm Cells

Viability staining was performed on cells resuspended from biofilms and directly on biofilms under each light and photosensitizing compound treatment condition using the LIVE/DEAD *Bac*Light viability kit (Invitrogen, Catalog #L7012) as described in [66] for use on *C. albicans* biofilms, and according to the manufacturer’s protocol. Briefly, the samples were incubated with 3 μL SYTO9 and 3 μL of propidium iodide in the dark at 30 °C for 20 min. Following incubation, the samples were imaged by fluorescence microscopy at 20× magnification with a green laser (GFP/green channel; 470 nm excitation wavelength) and a red laser (Texas Red/red channel; 585 nm excitation wavelength) using an EVOS Cell Imaging System (Life Technologies, Catalog #EVOS FL Cell Imaging System).

We note that due to an artifact of using this LIVE/DEAD stain when combined with certain photosensitizing compounds directly on biofilms, where the dead cells on the top of the biofilms appeared black (rather than red), likely due to their faster uptake of the photosensitizing compound over the LIVE/DEAD stain, we were unable to acquire valid images for certain treatment combinations when this stain was performed directly on biofilms. This artifact was not as readily apparent when using this LIVE/DEAD stain on cells resuspended from biofilms, and thus we were able to obtain valid images for all treatment combinations when this stain was performed on cells resuspended from biofilms.

### 2.6. Assessment of Cellular Morphologies of Biofilm Cells

Cells resuspended from biofilms under each light and photosensitizing compound treatment condition were imaged using brightfield microscopy at 20× magnification using an EVOS Cell Imaging System (Life Technologies, Catalog #EVOS FL Cell Imaging System) and the presence of hyphae, pseudohyphae, and yeast-form cells was qualitatively assessed.

## 3. Results

### 3.1. Effects of Red, Green, and Blue Visible Lights on C. albicans Biofilms

**Figure 2:**
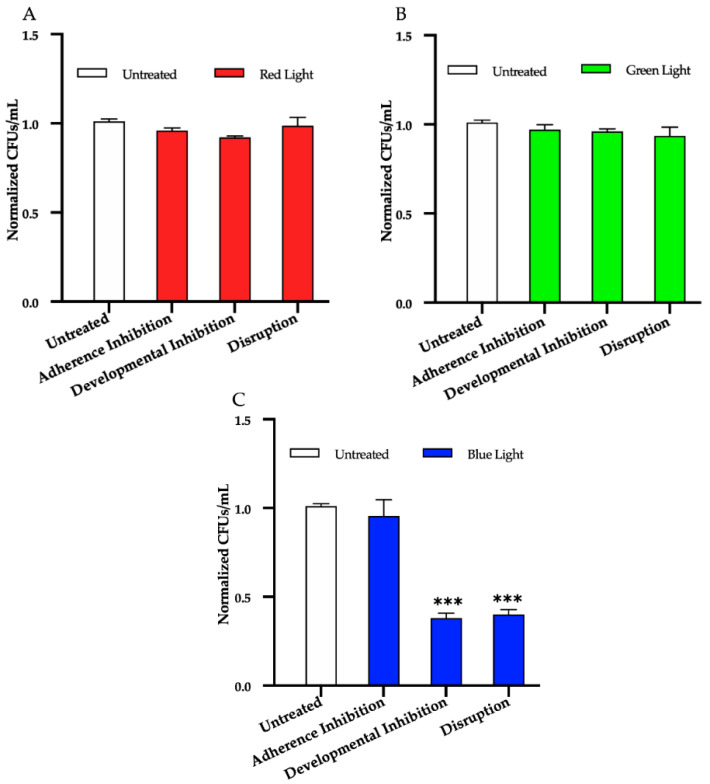
Effects of red, green, and blue visible lights on *C. albicans* biofilms. *C. albicans* biofilms were exposed individually to red, green, and blue lights in the adherence inhibition, developmental inhibition, and disruption biofilm assays. Colony forming units per 1mL (CFUs/mL) were measured to determine viable cell counts from the biofilms at the end of each biofilm assay. Effects of (**A**) red light, (**B**) green light, and (**C**) blue light in the three different biofilm assays are shown. Standard deviations are shown for each sample (*n* = 3). The average CFUs/mL of the untreated control samples for each assay were normalized to 1. Significance comparisons are relative to the untreated control and were determined using Student’s unpaired two-tailed *t*-tests assuming unequal variance for *p* ≤ 0.001 (***).

To determine the effects of red, green, and blue visible lights alone (i.e., without the addition of exogenous photosensitizing compounds), we first performed the three biofilm assays in the presence individually of red, green, and blue light treatments. We found that, compared to the untreated control, red and green lights alone had no effect on biofilm formation in any of the three biofilm assays (Figure 2A,B), and that blue light alone had no effect at inhibiting biofilm formation in the adherence inhibition assay (Figure 2C). Blue light alone, however, was highly effective at inhibiting *C. albicans* biofilm formation by ~65% in the developmental inhibition biofilm assay (*p* = 0.0005) and at disrupting mature biofilms by ~60% in the disruption biofilm assay (*p* = 0.0006) compared to the untreated control (Figure 2C).

### 3.2. Effects of Red, Green, and Blue Visible Lights in Combination with Exogenous Photosensitizing Compounds on C. albicans Biofilms

**Figure 3:**
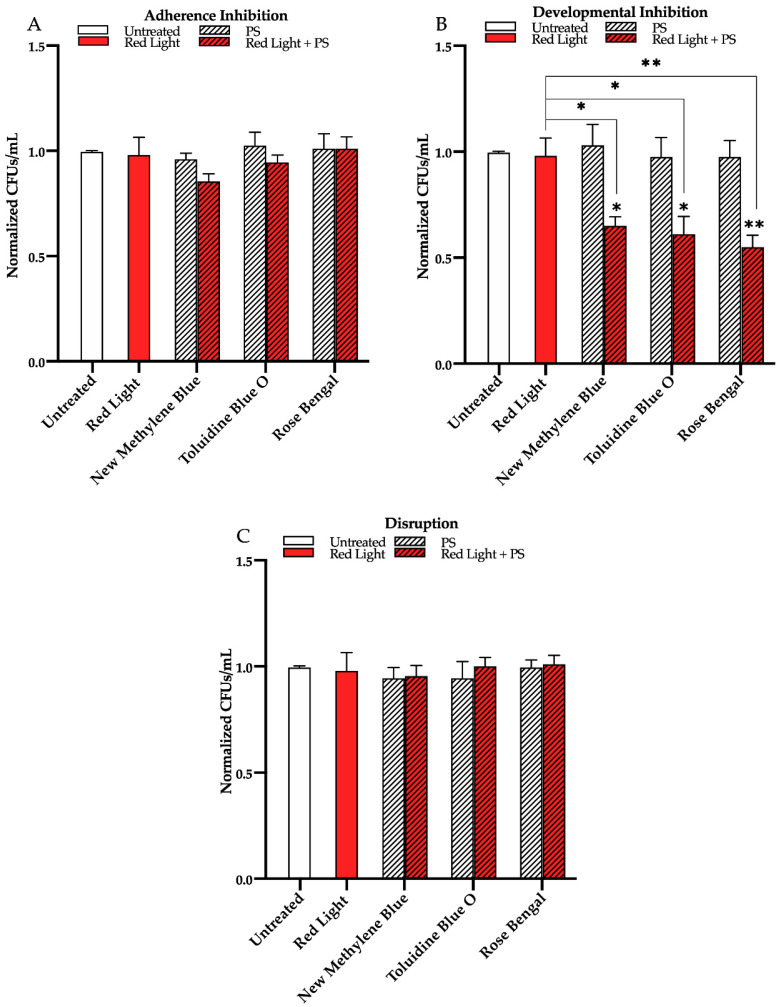
Effects of red visible light in combination with the photosensitizing compounds new methylene blue, toluidine blue O, and rose bengal on *C. albicans* biofilms. Effects of red light in combination with the photosensitizing compounds in the (**A**) adherence inhibition, (**B**) developmental inhibition, and (**C**) disruption biofilm assays. Untreated control (Untreated), red light alone (Red Light), photosensitizing compound alone (PS), and red light in combination with the photosensitizing compound (Red Light + PS) are shown. Colony forming units per 1mL (CFUs/mL) were measured to determine viable cell counts from the biofilms at the end of each biofilm assay. Standard deviations are shown for each sample (*n* = 3). The average CFUs/mL of the untreated control samples for each assay were normalized to 1. Significance comparisons are relative to the untreated control unless otherwise noted with significance bars and were determined using Student’s unpaired two-tailed t-tests assuming unequal variance for *p* ≤ 0.05 (*), and *p* ≤ 0.01 (**).

We next assessed the effects of red, green, and blue visible lights in combination with the commonly used exogenous photosensitizing compounds new methylene blue, toluidine blue O, and rose bengal on *C. albicans* biofilms. We found that, compared to the untreated control, red light alone, and each photosensitizing compound alone, red light in combination with any of the three photosensitizing compounds had no effects on biofilm formation in the adherence inhibition biofilm assay (Figure 3A). Red light, when combined with any of the three photosensitizing compounds in the developmental inhibition biofilm assay, however, was moderately effective at inhibiting *C. albicans* biofilm formation by ~30% when combined with new methylene blue (*p* = 0.03), ~40% when combined with toluidine blue O (*p* = 0.03), and ~45% when combined with rose bengal (*p* = 0.005) relative to the average of the untreated control, red light alone, and each photosensitizing compound alone (Figure 3B). We also assessed the effects of red light in combination with the three photosensitizing compounds on mature *C. albicans* biofilms in the disruption biofilm assay. We found that, compared to the untreated control, red light alone, and each photosensitizing compound alone, red light in combination with any of the three photosensitizing compounds had no effect on biofilm formation in the disruption biofilm assay (Figure 3C). Similar results were observed for red light in combination with these photosensitizing compounds on biofilm formation of two different *C. albicans* clinical isolates (see Figure S2 for results of the developmental inhibition biofilm assay on additional *C. albicans* strains).

**Figure 4:**
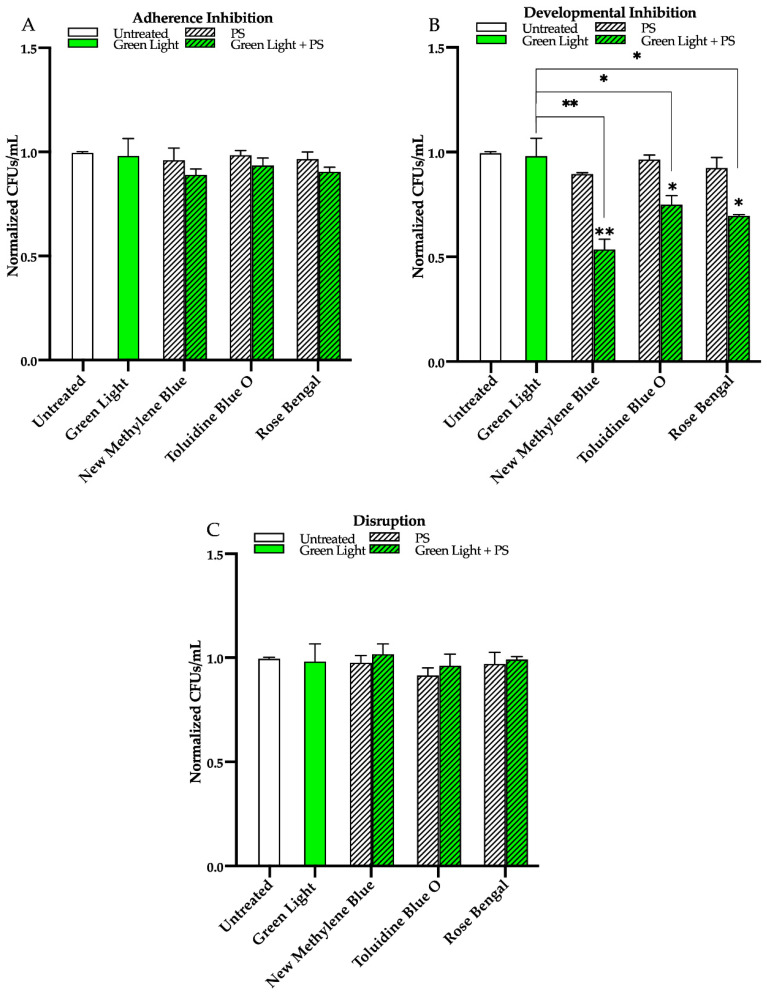
Effects of green visible light in combination with the photosensitizing compounds new methylene blue, toluidine blue O, and rose bengal on *C. albicans* biofilms. Effects of green light in combination with the photosensitizing compounds in the (**A**) adherence inhibition, (**B**) developmental inhibition, and (**C**) disruption biofilm assays. Untreated control (Untreated), green light alone (Green Light), photosensitizing compound alone (PS), and green light in combination with the photosensitizing compound (Green Light + PS) are shown. Colony forming units per 1 mL (CFUs/mL) were measured to determine viable cell counts from the biofilms at the end of each biofilm assay. Standard deviations are shown for each sample (*n* = 3). The average CFUs/mL of the untreated control samples for each assay were normalized to 1. Significance comparisons are relative to the untreated control unless otherwise noted with significance bars and were determined using Student’s unpaired two-tailed t-tests assuming unequal variance for *p* ≤ 0.05 (*), and *p* ≤ 0.01 (**).

Next, we found that compared to the untreated control, green light alone, and each photosensitizing compound alone, green light in combination with any of the three photosensitizing compounds had no effect on biofilm formation in the adherence inhibition biofilm assay (Figure 4A). Green light, when combined with any of the three photosensitizing compounds in the developmental inhibition biofilm assay, however, was moderately effective at inhibiting *C. albicans* biofilm formation by ~45% when combined with new methylene blue (*p* = 0.004), ~25% when combined with toluidine blue O (*p* = 0.02), and ~30% when combined with rose bengal (*p* = 0.03) relative to the average of the untreated control, green light alone, and each photosensitizing compound alone (Figure 4B). We also assessed the effects of green light in combination with the three photosensitizing compounds on mature *C. albicans* biofilms in the disruption biofilm assay. We found that, compared to the untreated control, green light alone, and each photosensitizing compound alone, green light in combination with any of the three photosensitizing compounds had no effect on biofilm formation in the disruption biofilm assay (Figure 4C). Similar results were observed for green light in combination with these photosensitizing compounds on biofilm formation of two different *C. albicans* clinical isolates (see Figure S3 for results of the developmental inhibition biofilm assay on additional *C. albicans* strains).

**Figure 5:**
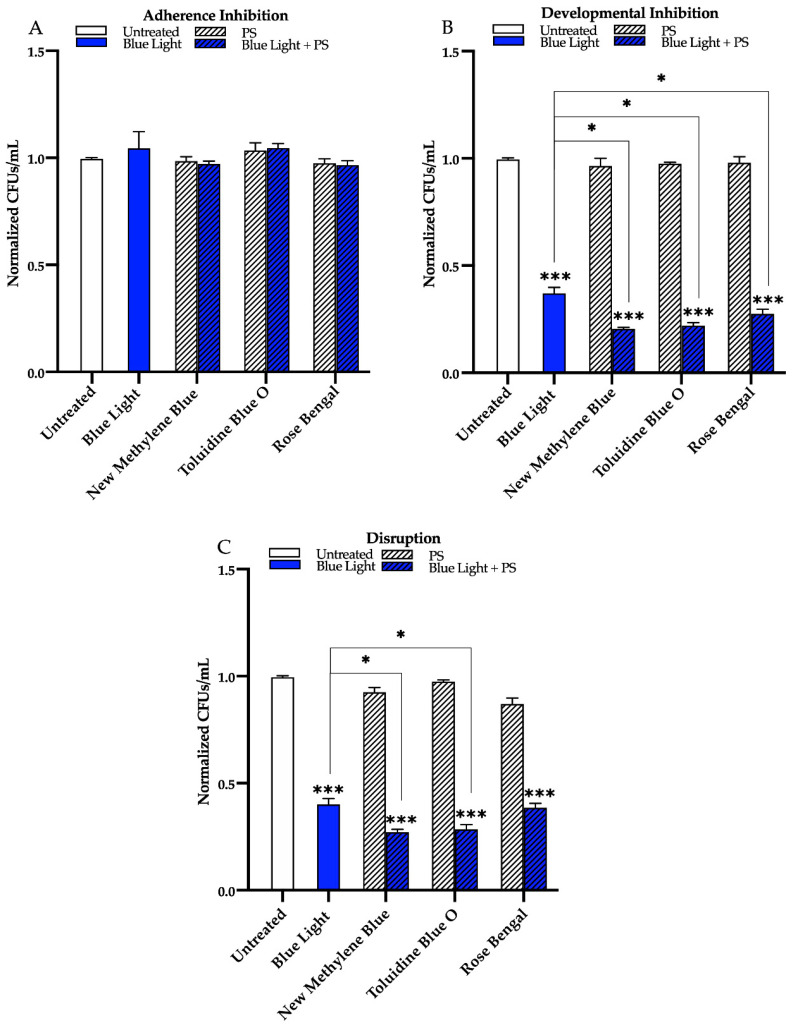
Effects of blue visible light in combination with the photosensitizing compounds new methylene blue, toluidine blue O, and rose bengal on *C. albicans* biofilms. Effects of blue light in combination with the photosensitizing compounds in the (**A**) adherence inhibition, (**B**) developmental inhibition, and (**C**) disruption biofilm assays. Untreated control (Untreated), blue light alone (Blue Light), photosensitizing compound alone (PS), and blue light in combination with the photosensitizing compound (Blue Light + PS) are shown. Colony forming units per 1 mL (CFUs/mL) were measured to determine viable cell counts from the biofilms at the end of each biofilm assay. Standard deviations are shown for each sample (*n* = 3). The average CFUs/mL of the untreated control samples for each assay were normalized to 1. Significance comparisons are relative to the untreated control unless otherwise noted with significance bars and were determined using Student’s unpaired two-tailed t-tests assuming unequal variance for *p* ≤ 0.05 (*), and *p* ≤ 0.001 (***).

We found that compared to the untreated control, blue light alone, and each photosensitizing compound alone, blue light in combination with any of the three photosensitizing compounds had no effect on biofilm formation in the adherence inhibition biofilm assay (Figure 5A). Blue light, when combined with any of the three photosensitizing compounds in the developmental inhibition biofilm assay, however, was highly effective at inhibiting *C. albicans* biofilm formation by ~80% when combined with new methylene blue (*p* = 0.0005), ~80% when combined with toluidine blue O (*p* = 0.0006), and ~70% when combined with rose bengal (*p* = 0.0008) relative to the average of the untreated control, and each photosensitizing compound alone (Figure 5B). Compared to the biofilm inhibitory effects of blue light alone, the combination of blue light with any of the three photosensitizing compounds in the developmental inhibition biofilm assay had an additive biofilm inhibitory effect of an additional 17% for new methylene blue (*p* = 0.01), 15% for toluidine blue O (*p* = 0.01), and 10% for rose bengal (*p* = 0.04) (Figure 5B). Similar results were observed for blue light in combination with these photosensitizing compounds on biofilm formation of two different *C. albicans* clinical isolates (see Figure S4A,B for results of the developmental inhibition biofilm assay on additional *C. albicans* strains).

Finally, we assessed the effects of blue light in combination with the three photosensitizing compounds on mature *C. albicans* biofilms in the disruption biofilm assay. We found that, compared to the untreated control, and each photosensitizing compound alone, blue light was effective at disrupting mature biofilms by ~75% when combined with new methylene blue (*p* = 0.0001), ~70% when combined with toluidine blue O (*p* = 0.0009), and ~60% when combined with rose bengal (*p* = 0.0009) (Figure 5C). Compared to the biofilm disruption effects of blue light alone, the combination of blue light with the photosensitizing compounds in the disruption biofilm assay had an additive biofilm disruption effect of an additional 14% for new methylene blue (*p* = 0.01) and 12% for toluidine blue O (*p* = 0.03) (Figure 5C). Compared to the biofilm disruption effect of blue light alone, no additive biofilm disruption effects were observed when blue light was combined with rose bengal (Figure 5C). Similar results were observed for blue light in combination with these photosensitizing compounds on biofilm formation of two different *C. albicans* clinical isolates, with the exception that for one of the clinical isolates (AR0761), an additive effect was also observed when blue light was combined with rose bengal in the disruption biofilm assay (see Figure S4C,D for results of the disruption biofilm assay on additional *C. albicans* strains).

As an independent assay for cell viability, we also performed LIVE/DEAD staining under the same conditions that we performed CFU determinations. We performed the LIVE/DEAD staining assay both on cells resuspended from biofilms and directly on biofilms under the different light and photosensitizing compound treatment conditions. Our cell viability staining results were consistent with our CFU determinations for all treatment conditions (see Figures S5–S8 for representative images from the LIVE/DEAD staining assay performed on cells resuspended from biofilms and Figures S9–S12 for representative images from the LIVE/DEAD staining assay preformed directly on biofilms). Lastly, we note that there were no qualitative differences in cellular morphologies (i.e., in the presence of hyphae, pseudohyphae, and yeast-form cells) between the untreated biofilms and biofilms treated with each of the three lights with or without the photosensitizing compounds (see Figure S13 for representative cellular morphology images for the treatment conditions with the largest antibiofilm effects for each light).

## 4. Discussion

Photodynamic therapy has been used to treat skin conditions for decades; however, its potential use as an antimicrobial strategy is only beginning to be recognized. Photodynamic therapy is thought to rely on the localized production of ROS that can have cytotoxic effects on the targeted cells. To comprehensively assess the potential utility of photodynamic therapy against *C. albicans* biofilms, we examined and compared the effects of red, green, and blue visible lights alone and in combination with the classic and commonly used photosensitizing compounds new methylene blue, toluidine blue O, and rose bengal. We note that the light intensities for each light we used in this study were similar, with red light at 6500 lux, green light at 6700 lux, and blue light at 5900 lux. Thus, the marginal differences in light intensities between the three lights did not seem to affect the results, especially given that blue light had the lowest light intensity but was the most effective against *C. albicans* biofilms. In fact, blue light alone was the only visible light tested that had antibiofilm properties on its own, where it markedly prevented biofilm formation when it was applied for 24 h throughout biofilm development, as well as markedly disrupting mature biofilms when it was applied for 24 h on a mature biofilm. Interestingly, when blue light alone was applied for just 90 min during the initial adherence stage of biofilm formation, it had no effects on inhibiting biofilm formation, indicating that prolonged exposure to blue light (i.e., longer than 90 min) is necessary for its antibiofilm potential. The combination of the photosensitizing compounds with red and green lights had moderate effects on preventing biofilm formation but had no effects on the initial 90 min adherence stage of biofilm formation or at disrupting mature biofilms. The fact that none of the light and photosensitizing compound combination treatments were effective at inhibiting biofilm formation during the 90 min adherence stage of biofilm formation was surprising. These findings indicate that exposure time to the light and photosensitizing compound treatments is an important factor in the antibiofilm efficacy of photodynamic therapy that may be related to the levels of ROS produced during the treatments. One hypothesis that could be tested in future studies is whether there is a direct relationship between light exposure time and ROS production.

Our findings indicate that the photosensitizing compounds were successful at sensitizing the biofilms to red and green lights when applied throughout biofilm development (i.e., for 24 h). The combination of the photosensitizing compounds with blue light had the most striking antibiofilm properties, where significant additive antibiofilm effects were observed in preventing biofilm formation and disrupting mature biofilms, significantly above those of blue light alone. Generally, these additive effects were especially noticeable when blue light was combined with new methylene blue and toluidine blue O, the two phenothiazinium salt photosensitizing compounds assessed. Overall, these findings indicate that photosensitizing compounds are effective at sensitizing the biofilm cells to light exposure, likely enhancing the production of ROS, and increasing cytotoxicity of the biofilm cells, with blue light plus new methylene blue, followed closely by blue light plus toluidine blue O, being the most effective treatment combinations against *C. albicans* biofilms.

Although the mechanism of action of blue light on microorganisms is not fully understood, a common hypothesis in the field is that exposure to blue light induces photoexcitation of naturally occurring endogenous photosensitizing compounds inside the microbial cells, such as flavoproteins and porphyrins, ultimately leading to ROS production and microbial cell death [40,44,45,67,68]. Indeed, one study has shown a clear correlation between porphyrin levels and microbial cell cytotoxicity upon exposure to blue light [69]. Consistent with this hypothesis, our work demonstrates that blue light alone induces *C. albicans* cell death within a biofilm, and that this effect is enhanced by the addition of photosensitizing compounds that lead to a further increase in the production of ROS.

In the context of biofilm infections, there are a number of drawbacks of traditional antifungal drug therapies that are overcome by the use of photodynamic antimicrobial therapies. First, the development of antifungal drug resistance after exposure to antifungal drugs can render traditional antifungal drug treatments virtually ineffective against biofilm infections. Given that photodynamic therapy generates ROS that affect multiple non-specific microbial targets simultaneously (e.g., causing lipid peroxidation, nucleic acid oxidation, and protein oxidation), it is unlikely that antimicrobial resistance to photodynamic therapy could be developed, and antimicrobial resistance to photodynamic therapy has not been reported to date [70,71,72]. Second, antifungal drugs, especially the polyenes (e.g., amphotericin B), have significant toxicities to human cells and are typically administered systemwide (e.g., intravenously) [11]. Photodynamic therapy utilizes non-toxic photosensitizing compounds combined with visible lights that pose little toxicity concerns to humans [23,25]. In addition, photodynamic therapy can be spatially confined to the infection area, thus limiting exposure of human cells to the treatment, and eliminating the toxicities associated with antifungal drugs administered systemwide. Third, antifungal drugs fail to penetrate into the lower levels of mature biofilms due to high microbial cell densities and the presence of the extracellular matrix, which has been shown to sequester antifungal drugs [73,74,75]. When photodynamic therapy is applied directly to the biofilm and ROS are produced, the small sizes of the ROS molecules should allow them to be easily transported into the lower levels of the biofilm via simple and/or facilitated diffusion, and ROS should be less likely to be sequestered by the extracellular matrix [12,76]. We note, however, that the physiological effects of photodynamic therapy on the extracellular matrix of biofilms have not been directly studied to date and are an area of interest for future studies in the field. Fourth, in order to effectively treat a biofilm infection, understanding the microbial composition of the biofilm is important in administering effective antimicrobial drug treatments. The majority of biofilm infections are not caused by a single microbial species, but are rather polymicrobial in nature, even containing microbial species that span different phylogenetic kingdoms, such as bacteria and fungi [4,77,78]. Studies have shown that polymicrobial biofilms are often much more resistant to antimicrobial drugs than single-species biofilms and are thus extremely challenging to treat [79]. Photodynamic therapy bypasses the need to know what microbial species are present in a polymicrobial biofilm infection because it has broad-spectrum antimicrobial efficacy, and has been shown to be effective against bacteria and fungi, even within polymicrobial biofilms [34,40,80,81,82,83,84,85]. Lastly, the mechanisms of action of almost all existing antimicrobial drugs (e.g., antibiotics and antifungals) target microbial metabolic processes, and thus require that the microbial cells are metabolically active in order to be effective [86,87,88,89,90]. This requirement poses significant inconsistencies in antimicrobial drug effectiveness in biofilms, where heterogeneous cell populations are located throughout the biofilm architecture with different levels of metabolic activity [74,91,92]. In addition, metabolically dormant phenotypic microbial cell variants within mature biofilms, called persister cells, are particularly difficult to eradicate with traditional antimicrobial drugs [74,88,93,94,95]. Photodynamic therapy, which uses ROS to kill microbial cells, does not require that the microbial cells are metabolically active, and there is some evidence to suggest that photodynamic therapy is effective against bacterial persister cells [25,96].

Given that there are only three major classes of antifungal drugs that are currently used to treat invasive fungal infections in humans, and that it has been a challenge to develop new and effective antifungal drugs, especially with efficacy against biofilms, there is a significant unmet medical need for new antifungal therapeutic strategies. Our work adds to the existing body of literature demonstrating that photodynamic therapy has the potential to be a clinically useful non-drug therapeutic strategy that is highly effective against *C. albicans* biofilms that could dramatically change the way we treat infectious diseases. Based on the present study as well as others in the field, photodynamic therapy shows excellent potential as a treatment approach for biofilm and other chronic infections. To date, most discussed clinical applications of photodynamic therapy for the treatment of infections are largely in the dermatology field, where photodynamic therapy could be applied to local infections on the skin using topical photosensitizing compounds and localized light exposure [97]. However, there are many other applications for photodynamic therapy that also show potential, such as its use in dentistry to treat persistent endodontic infections, such as periodontitis, peri-implantitis, and lesions from caries [98,99,100]. Despite its clear potential, the clinical use of photodynamic therapy to treat infections is still in its early stages and has not advanced as rapidly as other antimicrobial therapies. This is largely due to certain major limitations of its use, such as the fact that it needs to be applied locally and to areas of the body that can be accessed by light; thus its use against systemic infections is less likely to be feasible [97]. Another major limitation is that photodynamic therapy has not yet been standardized with clear and well-defined clinical parameters for the treatment of patients with infections. For example, we do not yet have defined effective dosages of photosensitizing compounds and we do not yet have standardized defined parameters for the duration of light exposure to be used in the treatment of specific types of infections [97]. Nonetheless, we believe that photodynamic therapy has great potential for clinical use in the treatment of localized infections, and its limitations in regard to standardizations should be overcome in the future with the development of defined clinical protocols.

## Data Availability

Data is contained within the article and Supplementary Material.

## Supplementary Materials

The following are available online at https://www.mdpi.com/2076-2607/9/3/500/s1: Figure S1. Chemical structures of the photosensitizing compounds used in these studies. (A) New methylene blue, (B) toluidine blue O, and (C) rose bengal are shown. Figure S2. Effects of red visible light in combination with the photosensitizing compounds new methylene blue, toluidine blue O, and rose bengal on biofilms formed by additional *C. albicans* strains. Effects of red light in combination with the photosensitizing compounds on the clinical isolates (A) SC5314 and (B) AR0761 in the developmental inhibition biofilm assay. Untreated control (Untreated), red light alone (Red Light), photosensitizing compound alone (PS), and red light in combination with the photosensitizing compound (Red Light + PS) are shown. Colony forming units per 1 mL (CFUs/mL) were measured to determine viable cell counts from the biofilms at the end of each biofilm assay. Standard deviations are shown for each sample (*n* = 3). The average CFUs/mL of the untreated control for each assay were normalized to 1. Significance comparisons are relative to an untreated control unless otherwise noted with significance bars and were determined using Student’s unpaired two-tailed t-tests assuming unequal variance for *p* ≤ 0.01 (**), and *p* ≤ 0.001 (***). Figure S3. Effects of green visible light in combination with the photosensitizing compounds new methylene blue, toluidine blue O, and rose bengal on biofilms formed by additional *C. albicans* strains. Effects of green light in combination with the photosensitizing compounds on the clinical isolates (A) SC5314 and (B) AR0761. Untreated control (Untreated), green light alone (Green Light), photosensitizing compound alone (PS), and green light in combination with the photosensitizing compound (Green Light + PS) are shown. Colony forming units per 1 mL (CFUs/mL) were measured to determine viable cell counts from the biofilms at the end of each biofilm assay. Standard deviations are shown for each sample (*n* = 3). The average CFUs/mL of the untreated control for each assay were normalized to 1. Significance comparisons are relative to an untreated control unless otherwise noted with significance bars and were determined using Student’s unpaired two-tailed t-tests assuming unequal variance for *p* ≤ 0.05 (*), *p* ≤ 0.01 (**), and *p* ≤ 0.001 (***). Figure S4. Effects of blue visible light in combination with the photosensitizing compounds new methylene blue, toluidine blue O, and rose bengal on biofilms formed by additional *C. albicans* strains. Effects of blue light in combination with the photosensitizing compounds on the clinical isolates (A) SC5314 and (B) AR0761 in the developmental inhibition biofilm assay. Effects of blue light in combination with the photosensitizing compounds on the clinical isolates (C) SC5314 and (D) AR0761 in the disruption biofilm assay. Untreated control (Untreated), blue light alone (Blue Light), photosensitizing compound alone (PS), and blue light in combination with the photosensitizing compound (Blue Light + PS) are shown. Colony forming units per 1 mL (CFUs/mL) were measured to determine viable cell counts from the biofilms at the end of each biofilm assay. Standard deviations are shown for each sample (*n* = 3). The average CFUs/mL of the untreated control for each assay were normalized to 1. Significance comparisons are relative to an untreated control unless otherwise noted with significance bars and were determined using Student’s unpaired two-tailed t-tests assuming unequal variance for *p* ≤ 0.05 (*), and *p* ≤ 0.001 (***). Figure S5. Effects of red visible light in combination with the photosensitizing compound rose bengal on cell viability of cells resuspended from biofilms in the developmental inhibition biofilm assay. The viability of cells resuspended from biofilms was assessed using the LIVE/DEAD *Bac*Light viability kit, where green fluorescence indicates live cells and red fluorescence indicates dead cells. The samples were imaged by fluorescence microscopy at 20× magnification with a green laser (GFP/green channel) shown in the top panel, a red laser (Texas Red/red channel) shown in the middle panel, and overlayed shown in the bottom panel. Representative images are shown for the untreated control (Untreated), red light alone (Red Light), rose bengal photosensitizing compound alone (Rose Bengal), and red light in combination with rose bengal photosensitizing compound (Red Light + Rose Bengal). Scale bars represent 200 μm. Figure S6. Effects of green visible light in combination with the photosensitizing compound new methylene blue on cell viability of cells resuspended from biofilms in the developmental inhibition biofilm assay. The viability of cells resuspended from biofilms was assessed using the LIVE/DEAD *Bac*Light viability kit, where green fluorescence indicates live cells and red fluorescence indicates dead cells. The samples were imaged by fluorescence microscopy at 20× magnification with a green laser (GFP/green channel) shown in the top panel, a red laser (Texas Red/red channel) shown in the middle panel, and overlayed shown in the bottom panel. Representative images are shown for the untreated control (Untreated), green light alone (Green Light), new methylene blue photosensitizing compound alone (New Methylene Blue), and green light in combination with new methylene blue photosensitizing compound (Green Light + New Methylene Blue). Scale bars represent 200 μm. Figure S7. Effects of blue visible light in combination with the photosensitizing compound new methylene blue on cell viability of cells resuspended from biofilms in the developmental inhibition biofilm assay. The viability of cells resuspended from biofilms was assessed using the LIVE/DEAD *Bac*Light viability kit, where green fluorescence indicates live cells and red fluorescence indicates dead cells. The samples were imaged by fluorescence microscopy at 20× magnification with a green laser (GFP/green channel) shown in the top panel, a red laser (Texas Red/red channel) shown in the middle panel, and overlayed shown in the bottom panel. Representative images are shown for the untreated control (Untreated), blue light alone (Blue Light), new methylene blue photosensitizing compound alone (New Methylene Blue), and blue light in combination with new methylene blue photosensitizing compound (Blue Light + New Methylene Blue). Scale bars represent 200 μm. Figure S8. Effects of blue visible light in combination with the photosensitizing compound new methylene blue on cell viability of cells resuspended from biofilms in the disruption biofilm assay. The viability of cells resuspended from biofilms was assessed using the LIVE/DEAD *Bac*Light viability kit, where green fluorescence indicates live cells and red fluorescence indicates dead cells. The samples were imaged by fluorescence microscopy at 20× magnification with a green laser (GFP/green channel) shown in the top panel, a red laser (Texas Red/red channel) shown in the middle panel, and overlayed shown in the bottom panel. Representative images are shown for the untreated control (Untreated), blue light alone (Blue Light), new methylene blue photosensitizing compound alone (New Methylene Blue), and blue light in combination with new methylene blue photosensitizing compound (Blue Light + New Methylene Blue). Scale bars represent 200 μm. Figure S9. Effects of red visible light in combination with the photosensitizing compound rose bengal on cell viability of biofilms in the developmental inhibition biofilm assay. The viability of biofilms was assessed using the LIVE/DEAD *Bac*Light viability kit, where green fluorescence indicates live cells and red fluorescence indicates dead cells. The samples were imaged by fluorescence microscopy at 20× magnification with a green laser (GFP/green channel) shown in the top panel, a red laser (Texas Red/red channel) shown in the middle panel, and overlayed shown in the bottom panel. Representative images are shown for the untreated control (Untreated), red light alone (Red Light), rose bengal photosensitizing compound alone (Rose Bengal), and red light in combination with rose bengal photosensitizing compound (Red Light + Rose Bengal). Scale bars represent 200 μm. Figure S10. Effects of green visible light on cell viability of biofilms in the developmental inhibition biofilm assay. The viability of biofilms was assessed using the LIVE/DEAD *Bac*Light viability kit, where green fluorescence indicates live cells and red fluorescence indicates dead cells. The samples were imaged by fluorescence microscopy at 20× magnification with a green laser (GFP/green channel) shown in the top panel, a red laser (Texas Red/red channel) shown in the middle panel, and overlayed shown in the bottom panel. Representative images are shown for the untreated control (Untreated), and green light alone (Green Light). Scale bars represent 200 μm. Figure S11. Effects of blue visible light on cell viability of biofilms in the developmental inhibition biofilm assay. The viability of biofilms was assessed using the LIVE/DEAD *Bac*Light viability kit, where green fluorescence indicates live cells and red fluorescence indicates dead cells. The samples were imaged by fluorescence microscopy at 20× magnification with a green laser (GFP/green channel) shown in the top panel, a red laser (Texas Red/red channel) shown in the middle panel, and overlayed shown in the bottom panel. Representative images are shown for the untreated control (Untreated), and blue light alone (Blue Light). Scale bars represent 200 μm. Figure S12. Effects of blue visible light on cell viability of biofilms in the disruption biofilm assay. The viability of biofilms was assessed using the LIVE/DEAD *Bac*Light viability kit, where green fluorescence indicates live cells and red fluorescence indicates dead cells. The samples were imaged by fluorescence microscopy at 20× magnification with a green laser (GFP/green channel) shown in the top panel, a red laser (Texas Red/red channel) shown in the middle panel, and overlayed shown in the bottom panel. Representative images are shown for the untreated control (Untreated), and blue light alone (Blue Light). Scale bars represent 200 μm. Figure S13. Assessment of cellular morphology of biofilm cells. Cells resuspended from biofilms were imaged by brightfield microscopy at 20× magnification. Biofilm cell morphologies consisting of hyphae, pseudohyphae and yeast-form cells were observed. Representative images are shown for the untreated control in the developmental inhibition biofilm assay (Developmental Inhibition Untreated), new methylene blue photosensitizing compound alone in the developmental inhibition biofilm assay (Developmental Inhibition New Methylene Blue), toluidine blue O photosensitizing compound alone in the developmental inhibition biofilm assay (Developmental Inhibition Toluidine Blue O), rose bengal photosensitizing compound alone in the developmental inhibition biofilm assay (Developmental Inhibition Rose Bengal), red light in combination with rose bengal photosensitizing compound in the developmental inhibition biofilm assay (Developmental Inhibition Red Light + Rose Bengal), green light in combination with new methylene blue photosensitizing compound in the developmental inhibition biofilm assay (Developmental Inhibition Green Light + New Methylene Blue), blue light in the developmental inhibition biofilm assay (Developmental Inhibition Blue Light), blue light in combination with new methylene blue photosensitizing compound in the developmental inhibition biofilm assay (Developmental Inhibition Blue Light + New Methylene Blue), blue light in the disruption biofilm assay (Disruption Blue Light), and blue light in combination with new methylene blue photosensitizing compound in the disruption biofilm assay (Disruption Blue Light + New Methylene Blue). Scale bar represents 200 μm.
