# Supplementary figures and images for: Visible Lights Combined with Photosensitizing Compounds Are Effective against Candida albicans Biofilms
Source: Microorganisms. 2021 Feb 26;9(3):500. doi: 10.3390/microorganisms9030500 (PMC7996876; doi:10.3390/microorganisms9030500)

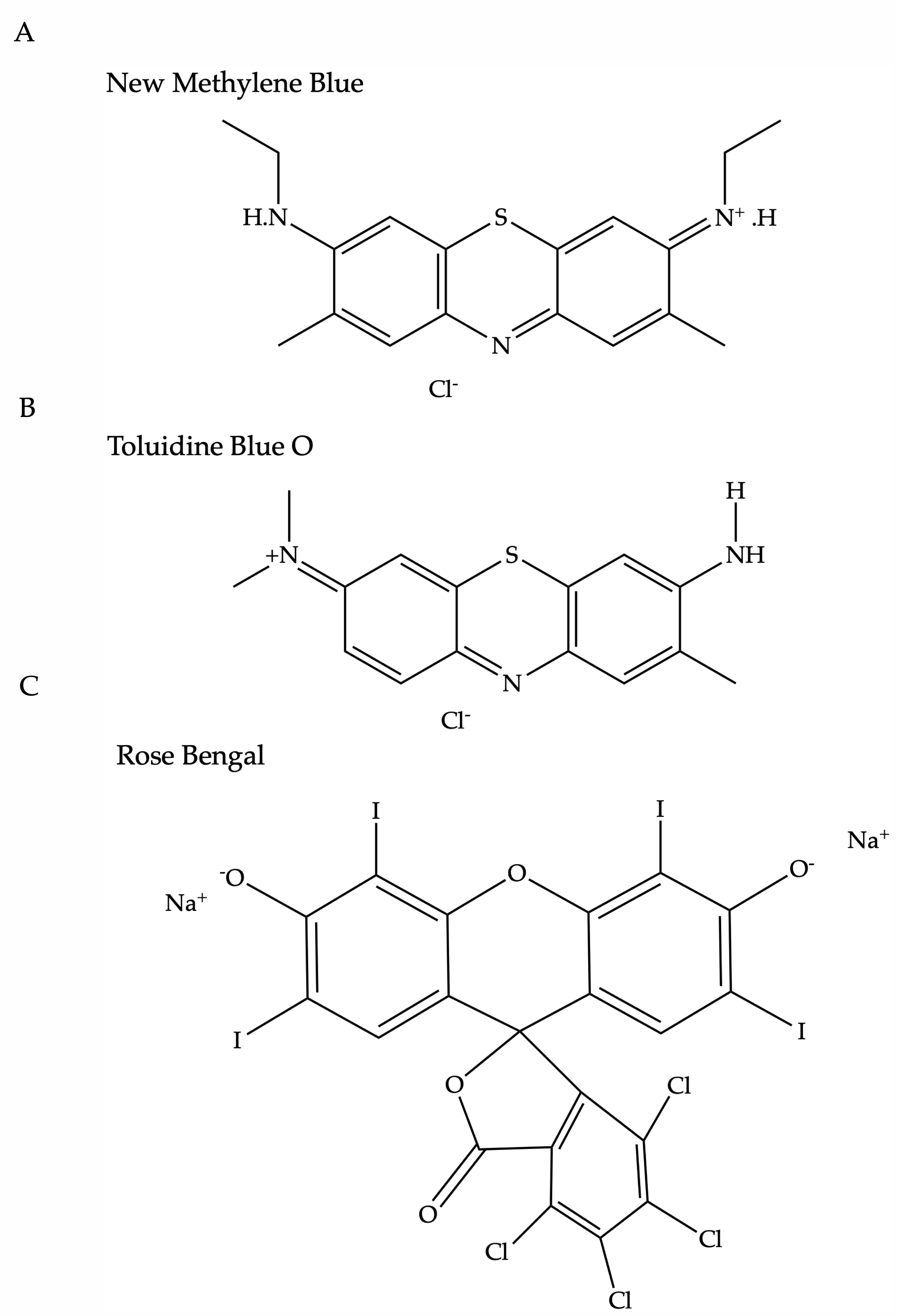

Supplement: Supplementary file 1 [file microorganisms-09-00500-s001.zip › Revised Figure S1 Final.tiff]

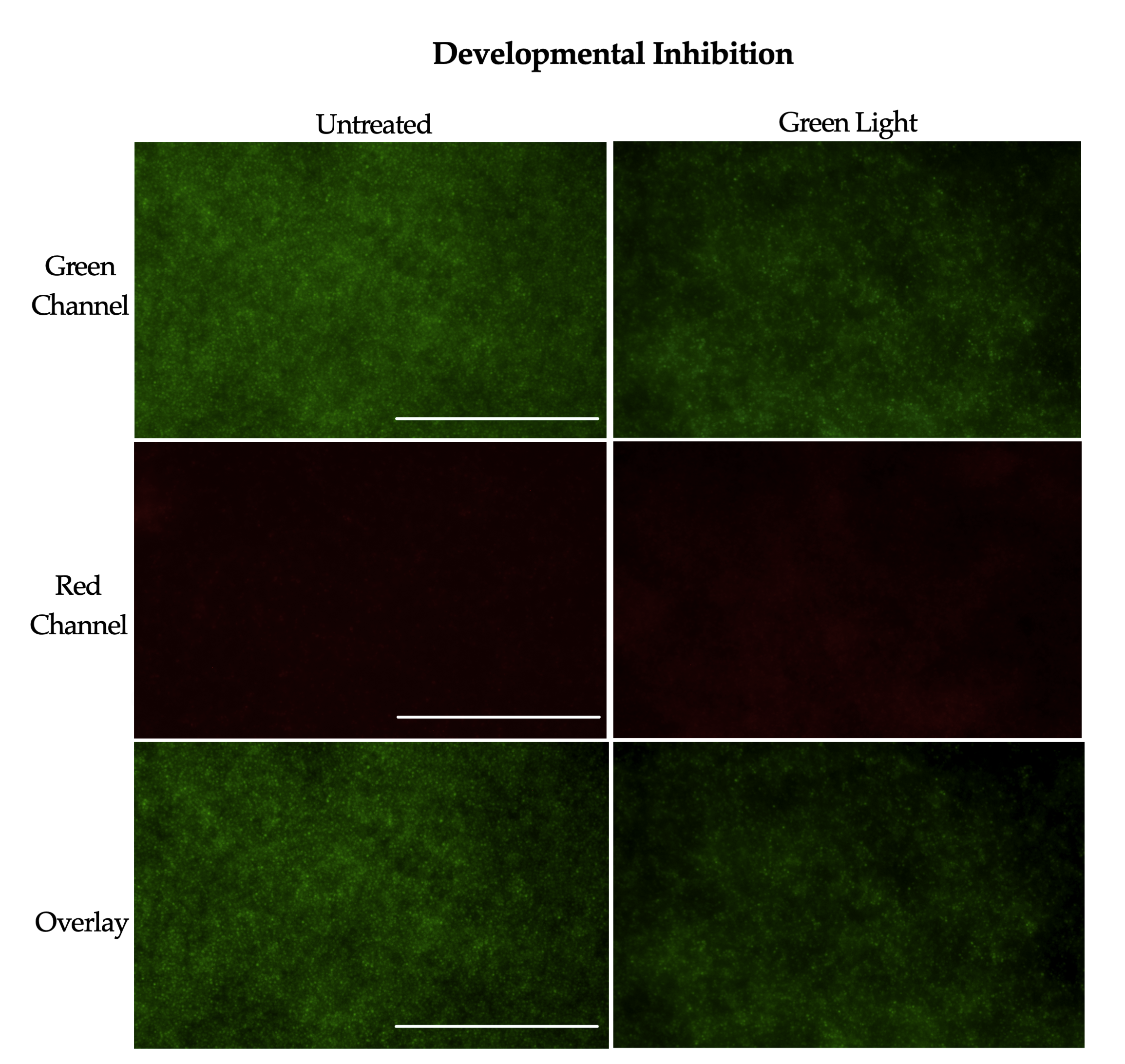

Supplement: Supplementary file 1 [file microorganisms-09-00500-s001.zip › Revised Figure S10 Final.tiff]

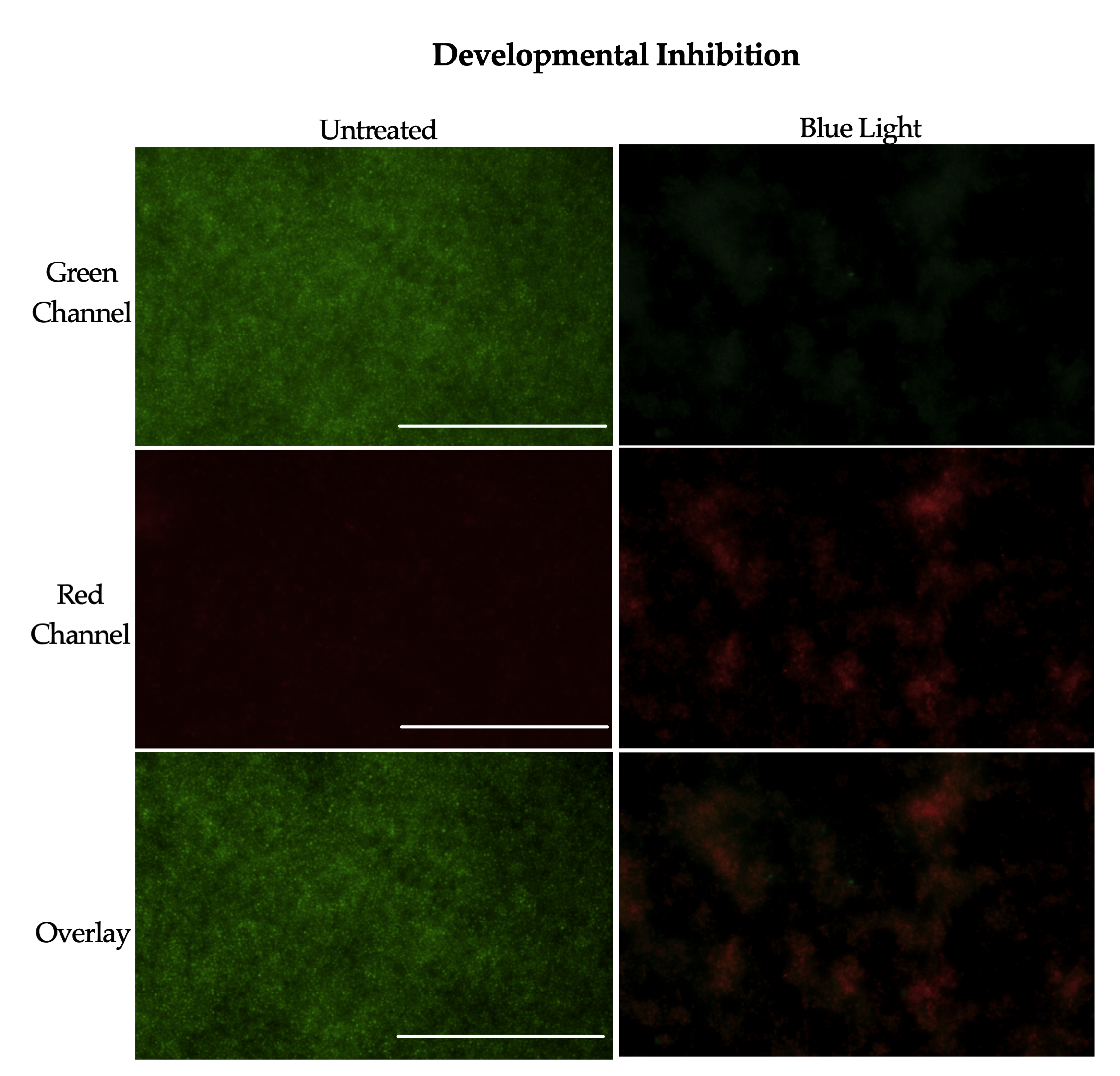

Supplement: Supplementary file 1 [file microorganisms-09-00500-s001.zip › Revised Figure S11 Final.tiff]

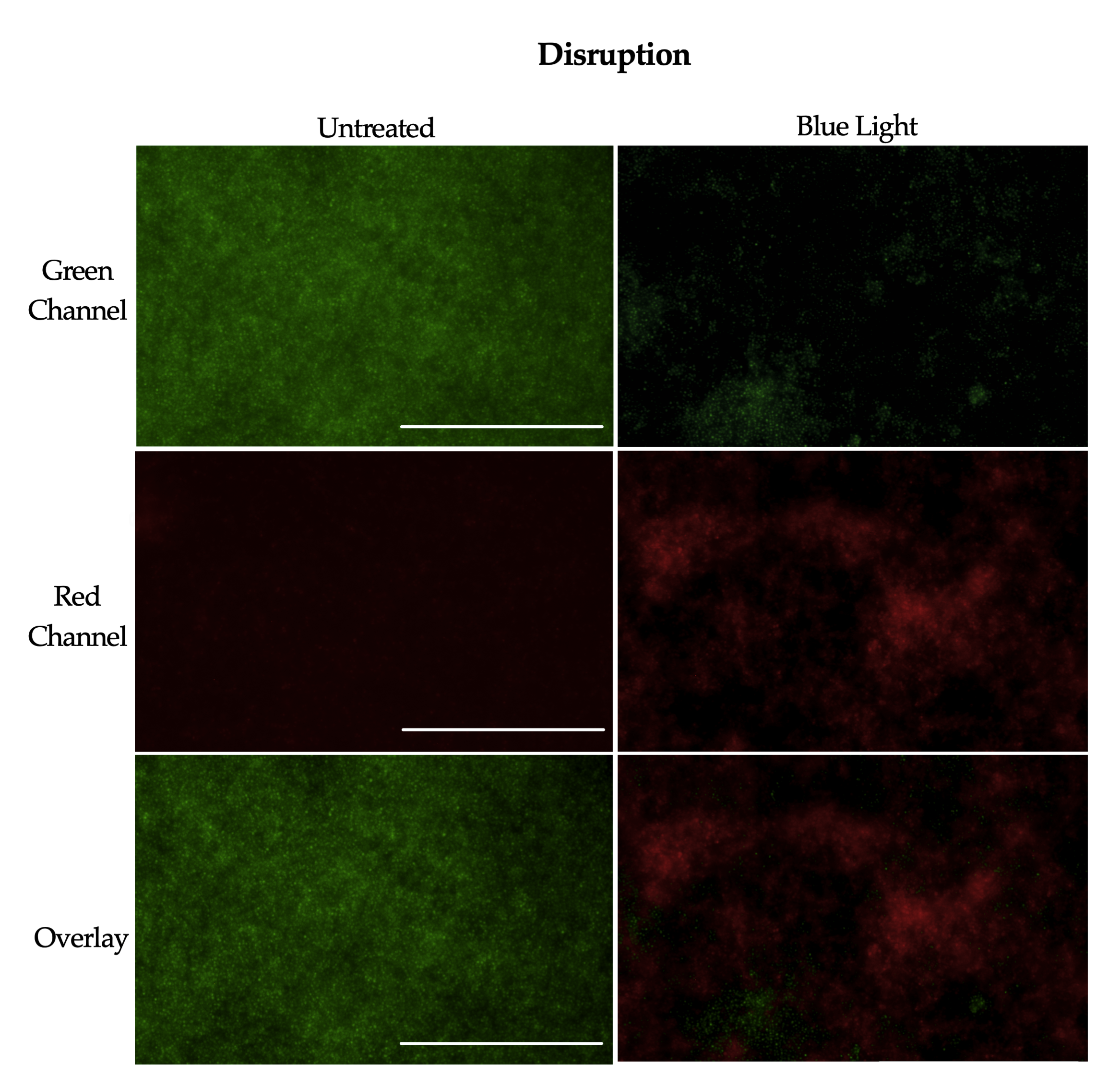

Supplement: Supplementary file 1 [file microorganisms-09-00500-s001.zip › Revised Figure S12 Final.tiff]

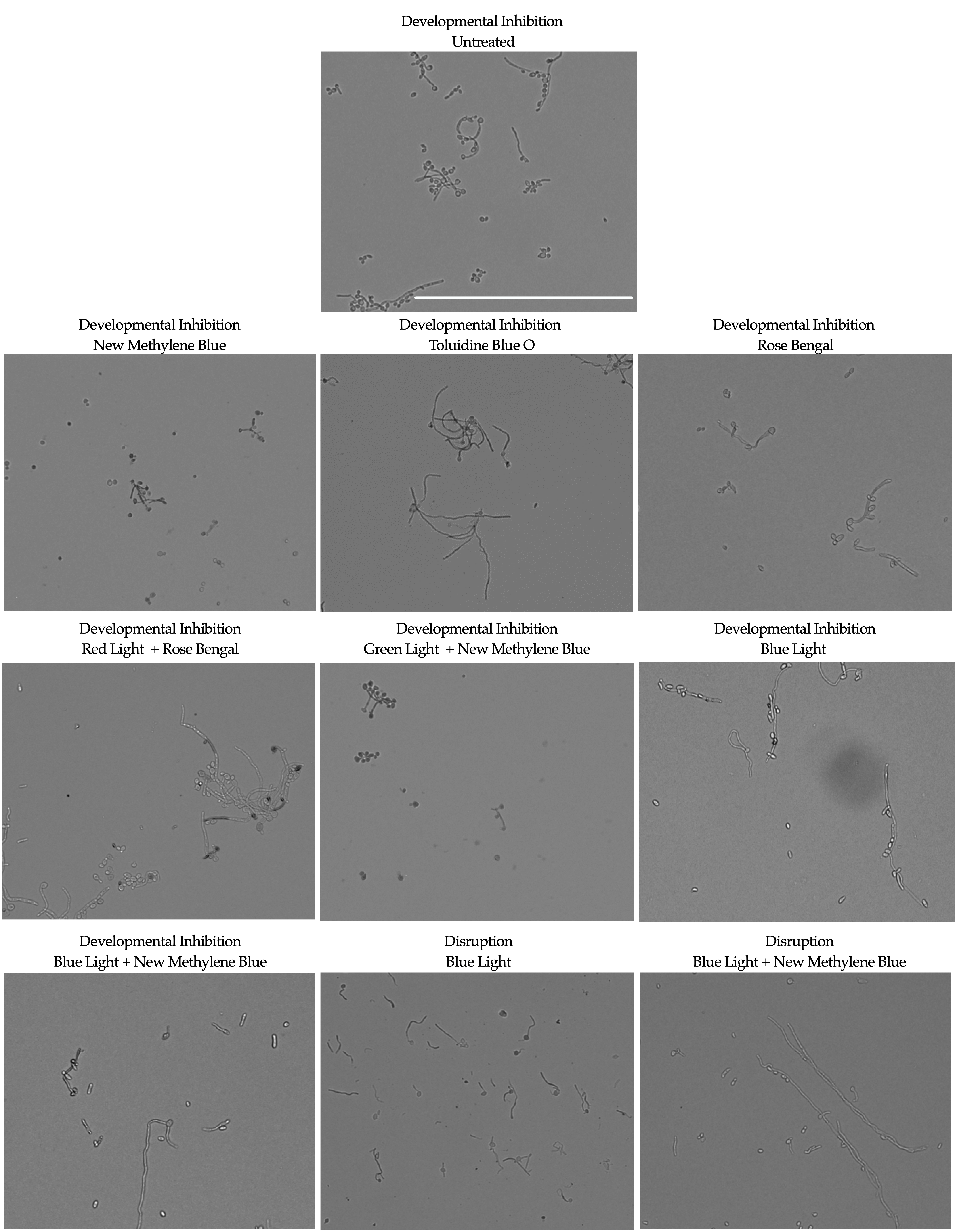

Supplement: Supplementary file 1 [file microorganisms-09-00500-s001.zip › Revised Figure S13 Final.tiff]

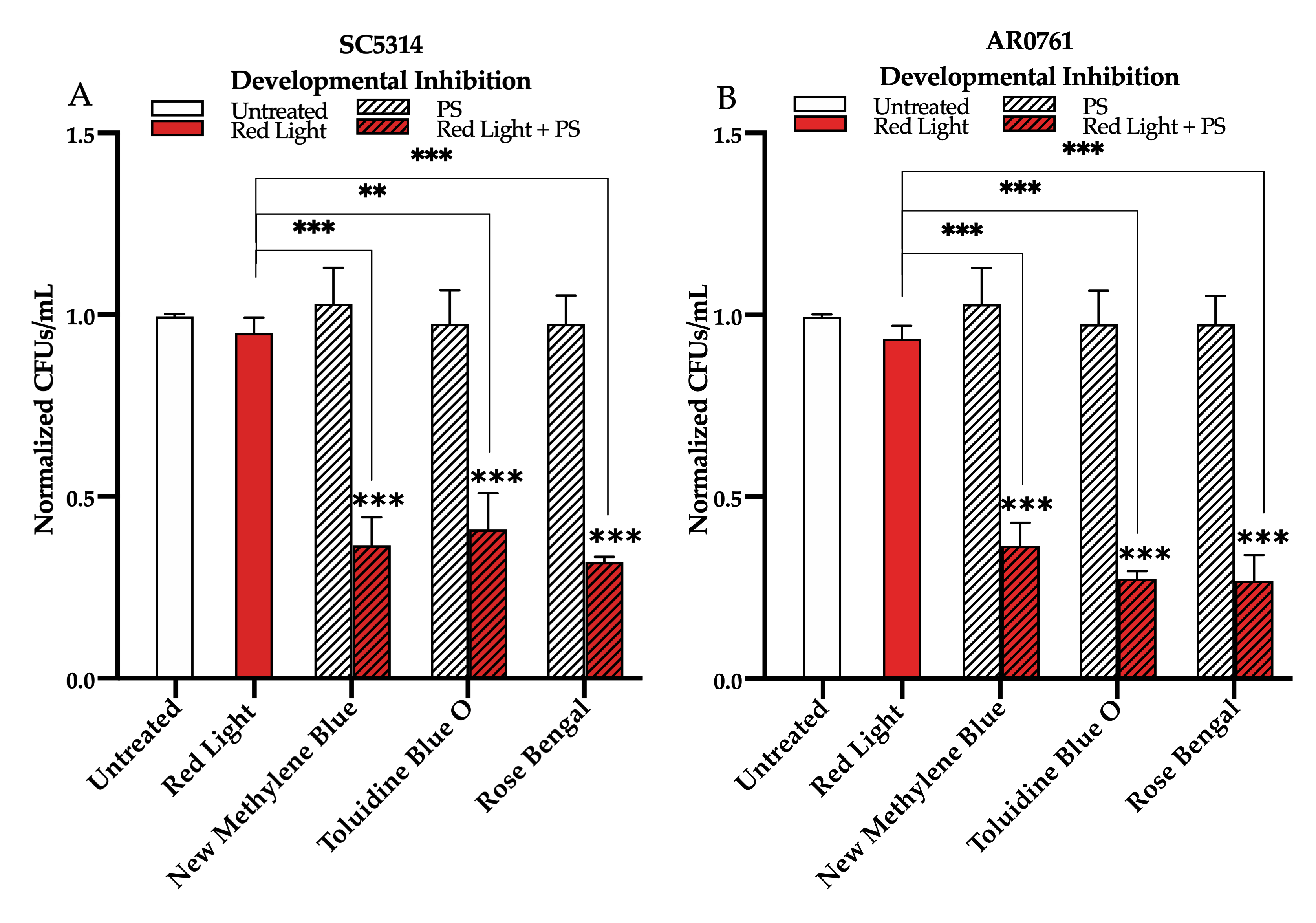

Supplement: Supplementary file 1 [file microorganisms-09-00500-s001.zip › Revised Figure S2 Final.tiff]

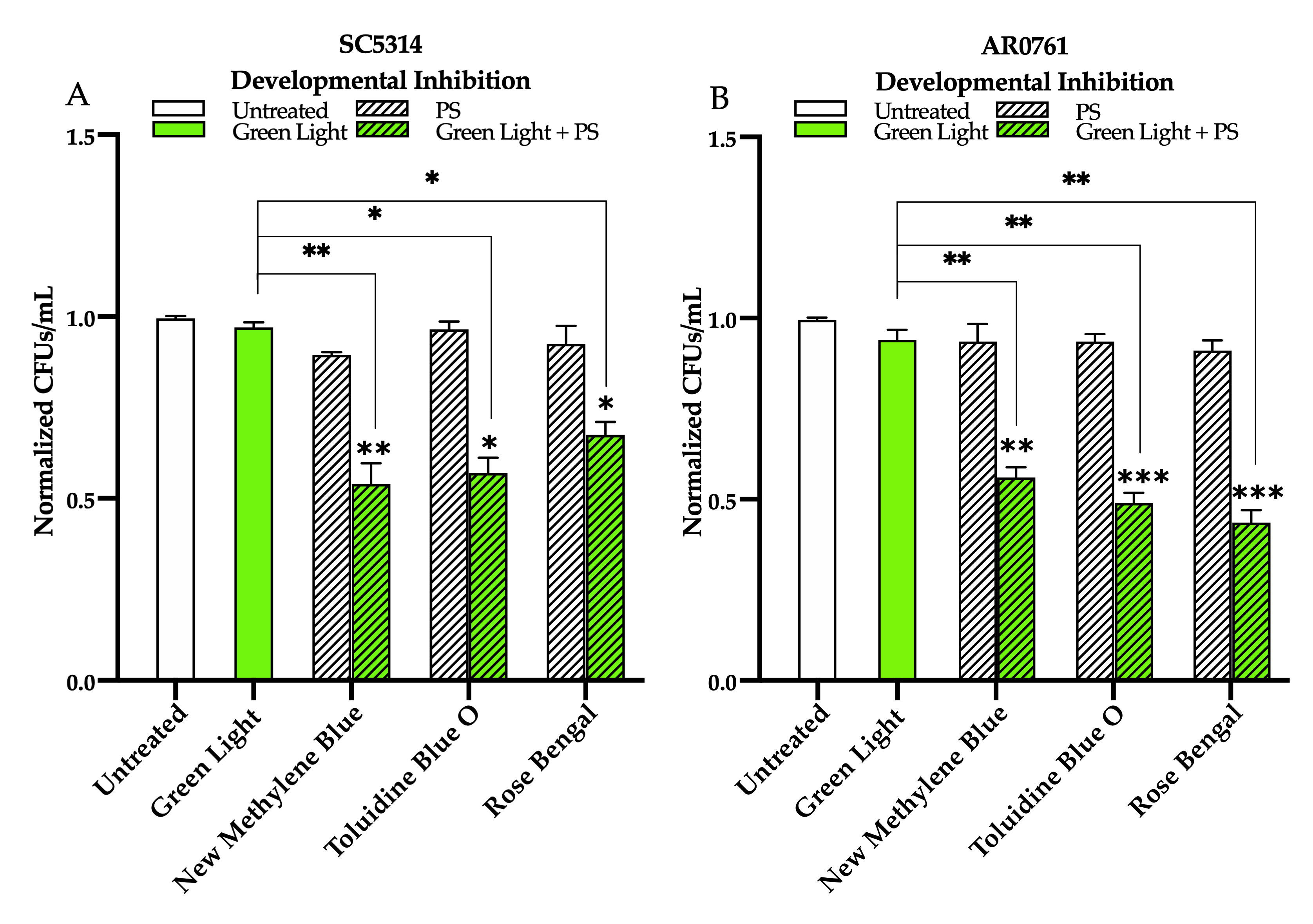

Supplement: Supplementary file 1 [file microorganisms-09-00500-s001.zip › Revised Figure S3 Final.tiff]

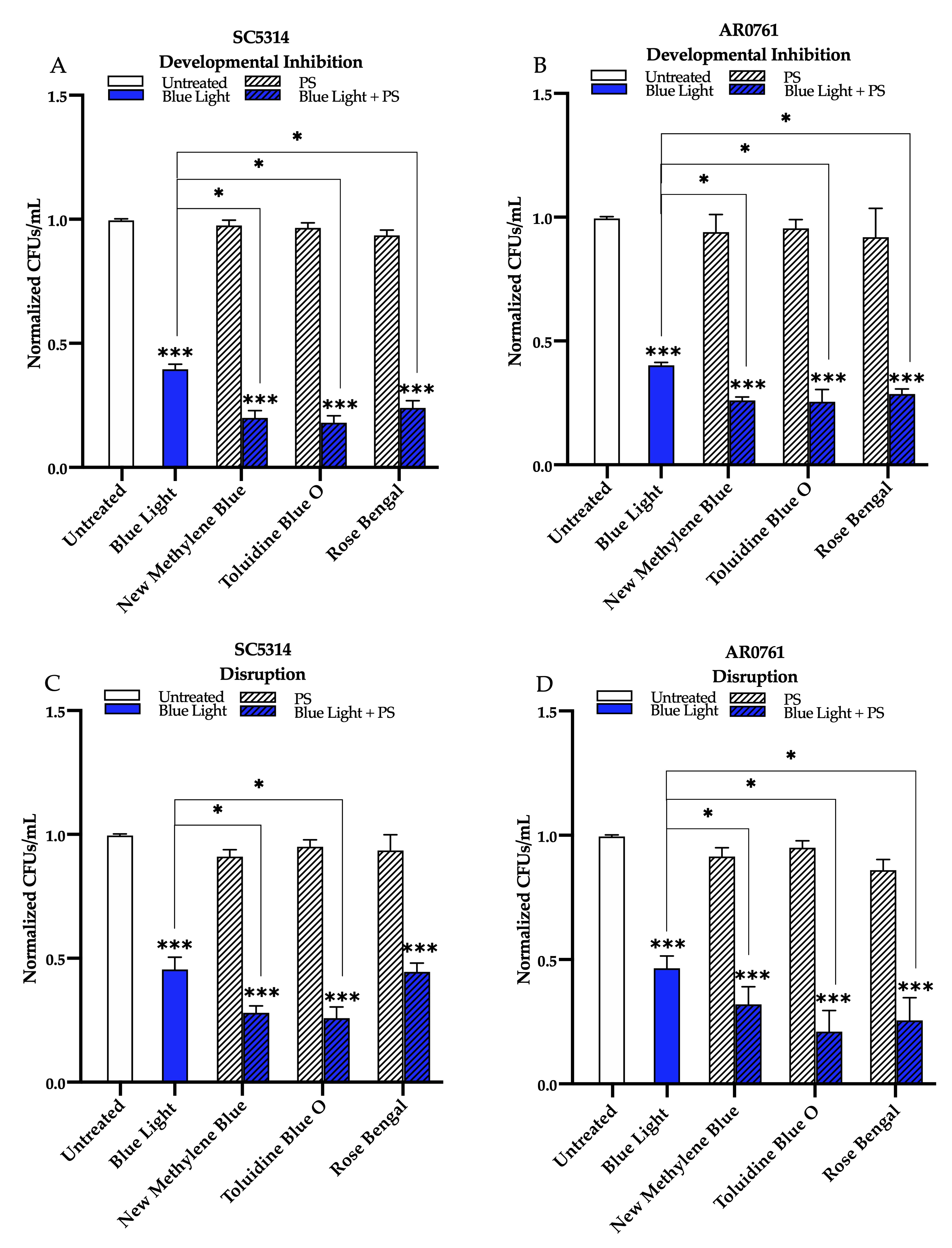

Supplement: Supplementary file 1 [file microorganisms-09-00500-s001.zip › Revised Figure S4 Final.tiff]

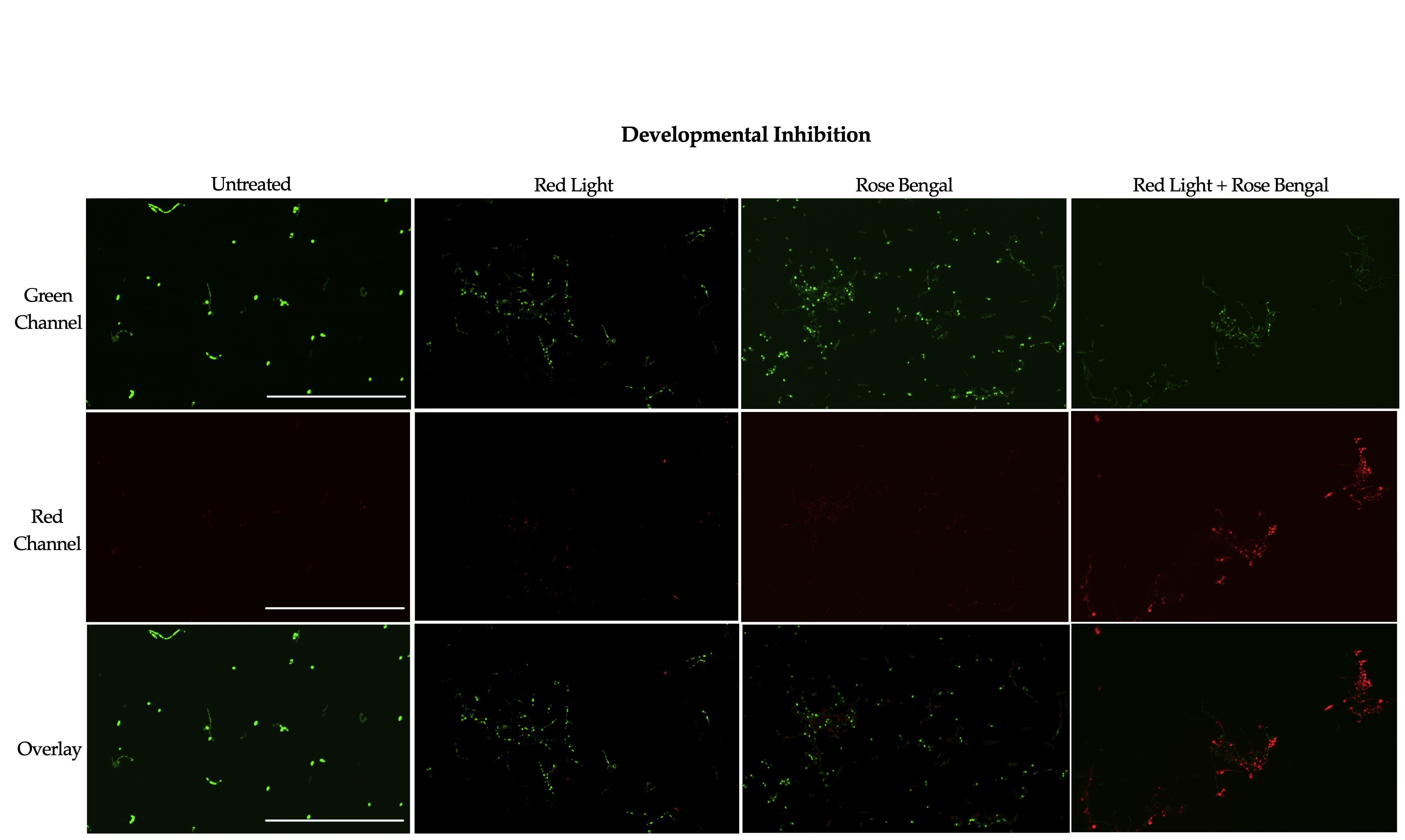

Supplement: Supplementary file 1 [file microorganisms-09-00500-s001.zip › Revised Figure S5 Final.tiff]

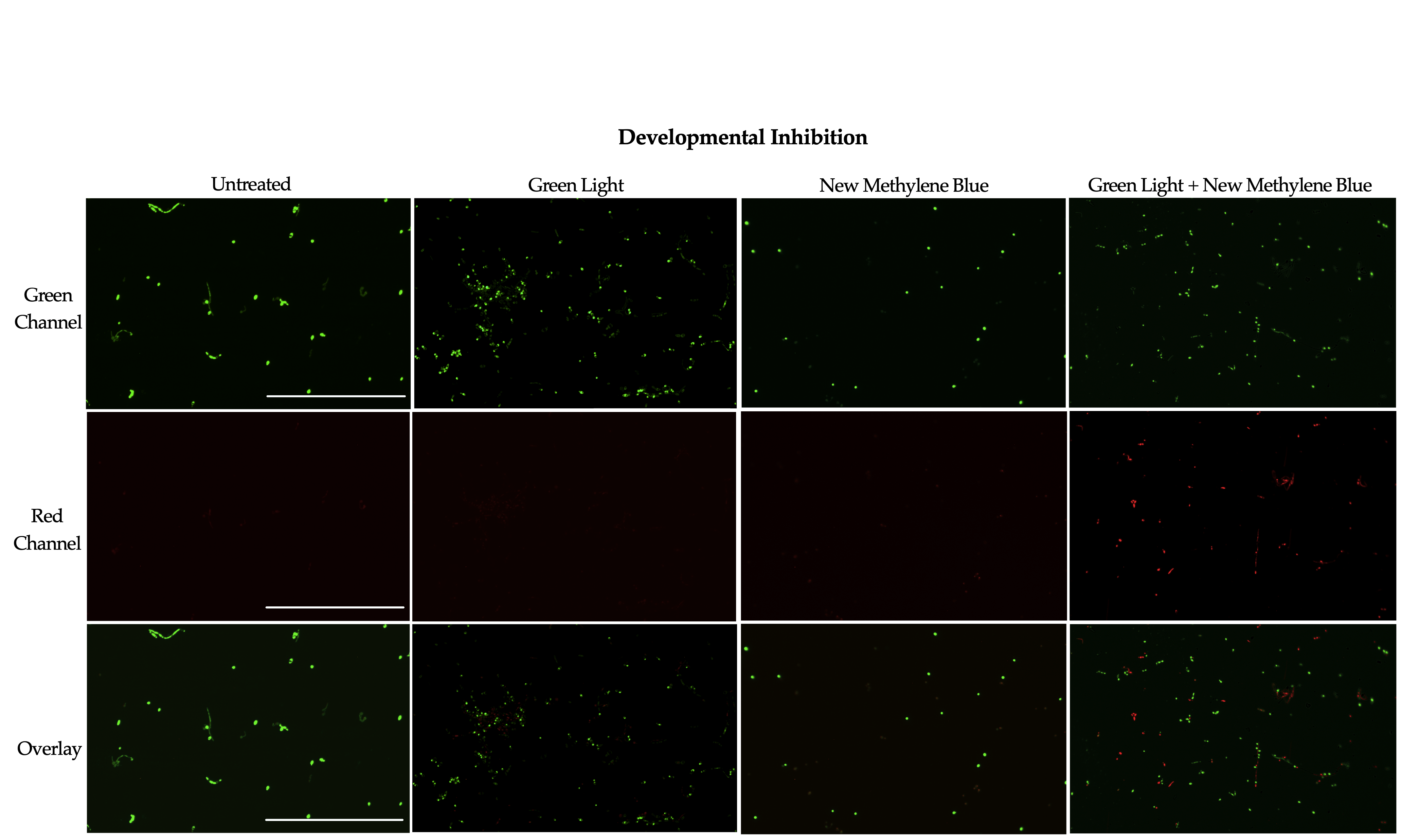

Supplement: Supplementary file 1 [file microorganisms-09-00500-s001.zip › Revised Figure S6 Final.tiff]

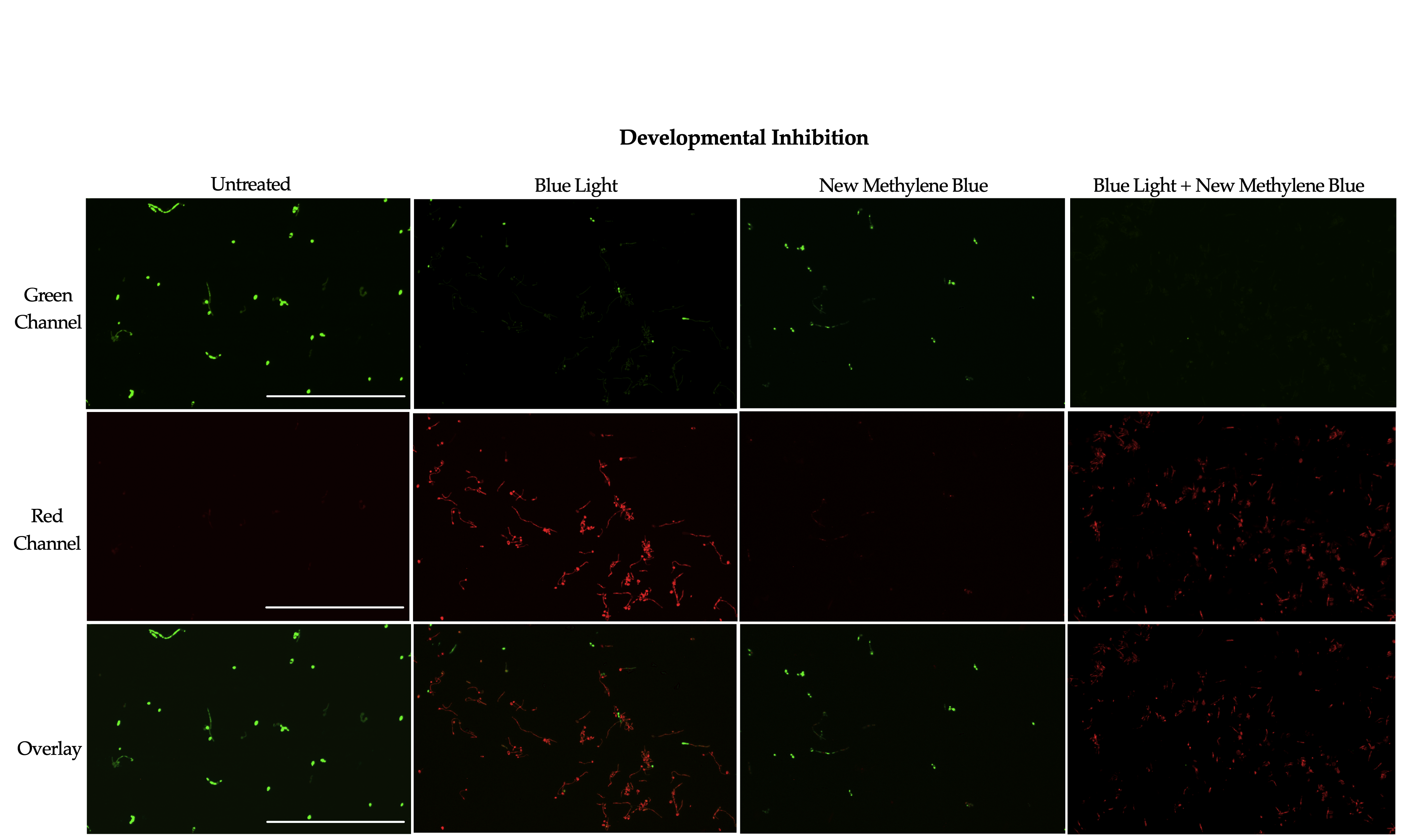

Supplement: Supplementary file 1 [file microorganisms-09-00500-s001.zip › Revised Figure S7 Final.tiff]

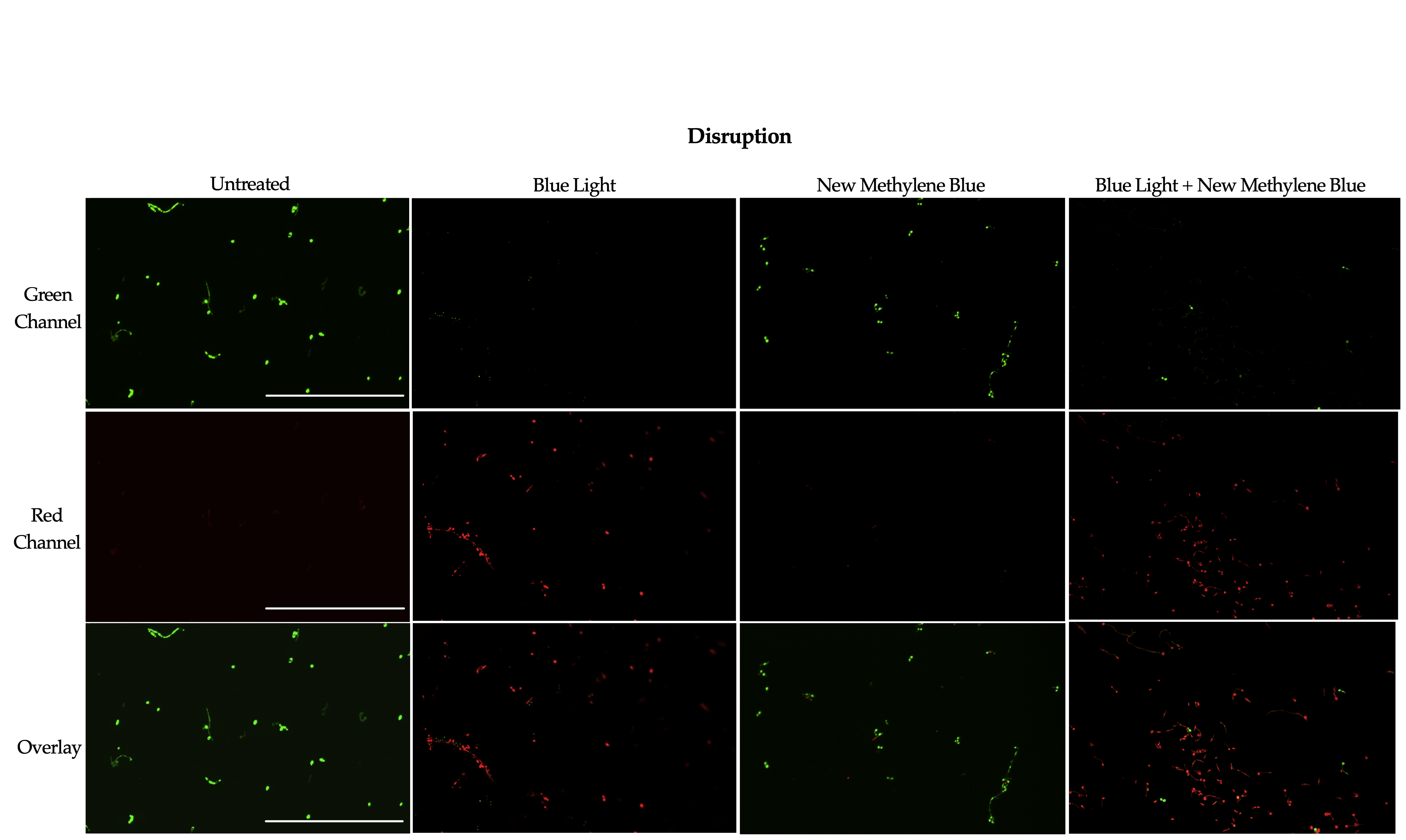

Supplement: Supplementary file 1 [file microorganisms-09-00500-s001.zip › Revised Figure S8 Final.tiff]

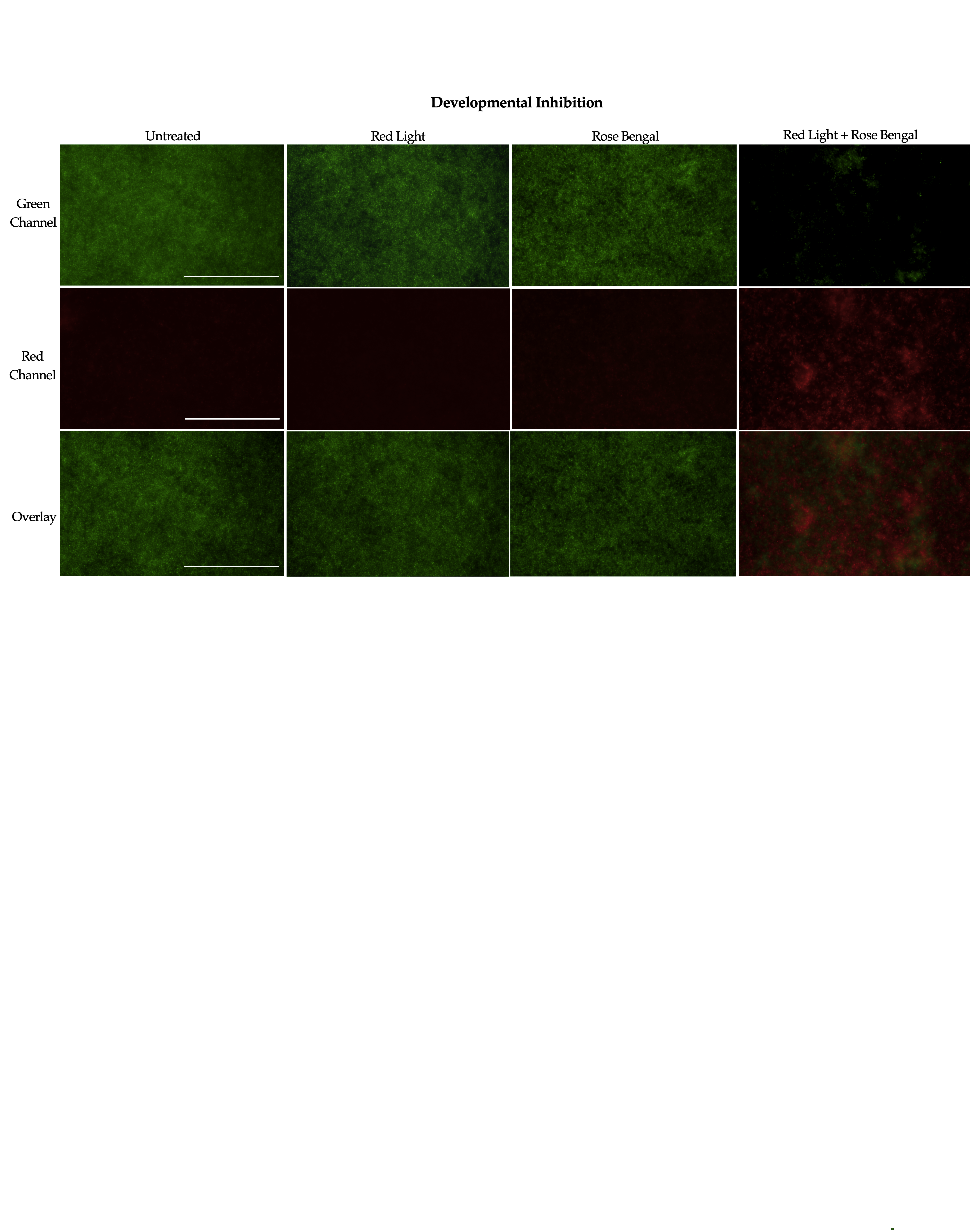

Supplement: Supplementary file 1 [file microorganisms-09-00500-s001.zip › Revised Figure S9 Final.tiff]
